# Engineering the First Chimeric Antibody in Targeting Intracellular PRL-3 Oncoprotein for Cancer Therapy in Mice

**DOI:** 10.18632/oncotarget.442

**Published:** 2012-02-27

**Authors:** Ke Guo, Jing Ping Tang, Li Jie, Abdul Qader O. Al-Aidaroos, Cheng William Hong, Cheng Peow Bobby Tan, Jung Eun Park, Leyon Varghese, Zhiwei Feng, Jianbiao Zhou, Wee Joo Chng, Qi Zeng

**Affiliations:** ^1^ Institute of Molecular and Cell Biology, A*STAR (Agency for Science, Technology and Research), 61 Biopolis Drive, Proteos, Singapore 138648, Republic of Singapore; ^2^ Cleveland Clinic Lerner College of Medicine, 9500 Euclid Avenue, Cleveland, OH 44195, USA; ^3^ Nanyang Technological University, School of Biological Sciences, 60 Nanyang Drive, Singapore 637551; ^4^ Cancer Science Institute of Singapore, National University of Singapore, 28 Medical Drive, Singapore 117456; ^5^ Department of Haematology-Oncology, National University Cancer Institute, Singapore National University Health System, 5 Lower Kent Ridge Road, S119074; ^6^ Department of Biochemistry, Yong Loo Lin School of Medicine, National University of Singapore, Singapore 119260, Republic of Singapore

**Keywords:** PRL-3 monoclonal antibody, PRL-3 mouse/human chimeric antibody, antibody therapy, intracellular oncoprotein

## Abstract

Antibodies are considered as ‘magic bullets’ because of their high specificity. It is believed that antibodies are too large to routinely enter the cytosol, thus antibody therapeutic approach has been limited to extracellular or secreted proteins expressed by cancer cells. However, many oncogenic proteins are localized within the cell. To explore the possibility of antibody therapies against intracellular targets, we generated a chimeric antibody targeting the intracellular PRL-3 oncoprotein to assess its antitumor activities in mice. Remarkably, we observed that the PRL-3 chimeric antibody could efficiently and specifically reduce the formation of PRL-3 expressing metastatic tumors. We further found that natural killer (NK) cells were important in mediating the therapeutic effect, which was only observed in a *nude* mouse model (T-cell deficient), but not in a Severe Combined Immunodeficiency’ (*scid*) mouse model (B- and T-cell deficient), indicating the anticancer effect also depends on host B-cell activity. Our study involving 377 *nude* and *scid* mice suggests that antibodies targeting intracellular proteins can be developed to treat cancer.

## INTRODUCTION

A century ago, Paul Ehrlich proposed the concept of antibodies as ‘magic bullets’, the use of therapeutic monoclonal antibodies (mAbs) against a number of disease targets validated this concept [1-3]. In general, antibody targeted therapy has much reduced toxicity than chemotherapy with small molecular weight chemical inhibitors [2]. Antibodies constitute the most rapidly growing class of human therapeutics and are ideal agents for recognizing and destroying malignant cells via the immune system. However, this therapeutic approach has been limited to surface or secreted proteins expressed by cancer cells [4], since antibody targeting of intracellular oncoproteins was previously thought to be unfeasible because of the intracellular location of the targets. As a consequence, a wide spectrum of intracellular oncoproteins remains unexplored in terms of antibody therapy approach. In 1978, Donato Alarcon-Segovia first discovered that antibodies could reach inside living cells [5]. Subsequently, the concept that intact antibodies are unable to gain entry into viable cells has been challenged experimentally as well as clinically. Over the past 2 decades, immunologists have shown that 1) autoantibodies found in the serum of autoimmune disease patients could bind to their respective intracellular antigens, and 2) immunologically mediated damage might occur when antibodies enter a living cell triggering apoptosis [6-8]. Consistently, it is demonstrated here that antibodies to an intracellular protein can exert therapeutic effects and suggest new insights to the use of therapeutic antibodies to include intracellular targets.

PRL-1 (phosphatase of regenerating liver-1), PRL-2, and PRL-3 represent an intriguing subgroup of the intracellular protein tyrosine phosphatases (PTP). Individual PRLs are overexpressed in a variety of cancer cell lines and cancer tissues when compared with their normal counterparts [9] and are reported to play multiple roles in cancer progression [10]. PRLs are intracellular C-terminally prenylated phosphatases. The localization of PRL-1 and PRL-3 to the inner leaflet of the plasma membrane and early endosomes was shown by electron microscopy (EM) immunogold labeling [11,12]. In contrast, the mutant forms of PRLs that lack the prenylation signal are localized in nuclei [13]. PRL-3 was first discovered as a metastasis-associated phosphatase in colorectal cancer metastasis, being consistently overexpressed in 100% of liver metastasis samples taken from 18 colorectal cancer (CRC) patients [14]. Overexpression of PRLs has been subsequently shown to have a causative role in promoting cancer metastases and they become potential targets for diverse cancer treatment [15]. As these phosphatases are intracellularly localized, the conventional approach using therapeutic antibodies would seem implausible. However, we recently reported an unexpected observation that mouse monoclonal antibodies (mAbs) against PRL-1 or PRL-3 were able to prevent the experimental metastasis of cancer cells overexpressing intracellular PRL-1 or PRL-3 [16].

To extend our earlier findings, in this current study, we expanded upon the reliability of such targeting strategy using a newly-generated mouse/human chimeric monoclonal PRL-3 antibody as a potential clinical therapeutic agent against cancer in five important aspects. Firstly, clinically-relevant chimeric antibodies instead of mouse antibodies were generated and characterized. Secondly, mice harboring naturally-occurring human cancer cells that express endogenous PRL-3 instead of Chinese hamster ovary (CHO) cells expressing exogenous PRL-3 were treated. Thirdly, it was shown that depletion of nature killer (NK) cells abolished the therapeutic response and aggravated tumor burden in mice. Fourthly, by using paired *nude* versus *scid* mouse models, a crucial role for B-cells in determining the outcome of our antibody therapy was identified. Finally, using the IVIS live imaging system, fluorescent labeled antibodies were used to track their tumor binding activities and their working models. An evidence-based concept is hereby proposed for a possible approach in targeting intracellular oncoproteins with antibody therapies. The results suggest that an evaluation of a wide spectrum of intracellular oncoproteins (such as phosphatases, kinases, transcription factors, and many others) as possible targets for anticancer therapy may be warranted.

## RESULTS

### Generation of PRL-3 mouse/human chimeric antibodies (clone #318)

**Figure 1:**
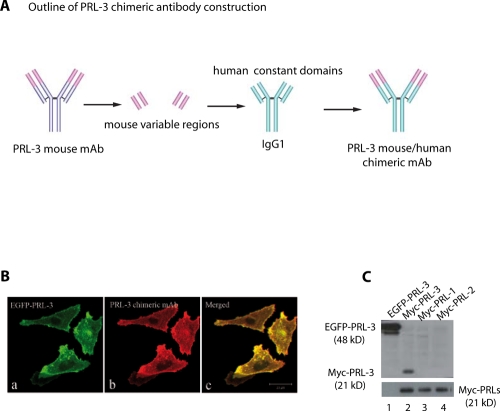
Generation of PRL-3 specific chimeric mAb **A**. A schematic diagram outlining the major steps of PRL-3 chimeric mAb construction. **B**. PRL-3 chimeric mAb recognizes EGFP-PRL-3 overexpressed in DLD-1 human colorectal cancer cells by indirect immunofluorescence: a, distribution of EGFP-PRL-3 in fixed DLD-1 cells (green); b, PRL-3 chimeric antibody and anti-human IgG Texas-Red to reveal the binding to PRL-3 protein; c, merged image. *Bar*, 20 μm. **C**. Cell lysate derived from DLD-1 cells overexpressing EGFP-PRL-3, and lysates derived from CHO stable cell lines overexpressing myc-PRL-3, myc-PRL-1, and myc-PRL-2 were analyzed by western blotting. PRL-3 chimeric antibody specifically recognized EGFP-PRL-3 (48 kDa, lane 1) and myc-PRL-3 (21 kDa, lane 2), but did not cross-react with myc-PRL-1 and myc-PRL-2 (lanes 3 and 4). Lower panel showed the equal loading of myc-PRL-3, -1, and -2 (21 kDa).

We previously reported that PRL-3 or PRL-1 mouse mAbs could specifically target their respective intracellular PRL-3 or PRL-1 phosphatase to inhibit cancer metastases in *nude* mice [16]. In an attempt to translate these laboratory findings to clinical setting, a mouse/human chimeric mAb against PRL-3 was engineered to minimize the potential antigenicity of the mouse mAb in human. Using recombinant DNA technology, we separately fused the constant domains of heavy or light chains of the human IgG1 molecule with the mouse variable regions of PRL-3 mAb (clone m318) by transgenic fusion of the immunoglobulin genes (Fig. 1A) [17,18]. The expression construct was transfected into Human Embryonic Kidney (HEK) 293T cells to produce recombinant PRL-3 chimeric mAb that was then harvested from the culture medium and further concentrated. The antigen-binding specificity of the PRL-3 chimeric antibody was well conserved as confirmed by indirect immuofluorescence on DLD-1 cells that overexpress exogenous EGFP-PRL-3 (Fig. 1B) and western blot analyses (Fig. 1C). The PRL-3 chimeric mAb (h318) specifically recognized both EGFP-PRL-3 (~48 kDa) and myc-PRL-3 (~21 kDa) (Fig. 1C, lane 1-2) but react with neither myc-PRL-1 nor myc-PRL-2 proteins (Fig. 1C, lane 3-4). A 50% cell Inhibitory Cytotoxic concentration (IC50) of the chimeric antibody was determined on a mouse melanoma B16F0 cell line that expresses endogenous PRL-3 protein. No cellular toxicity was observed in *in vitro* culture system even at concentrations as high as 40 μg/ml (Fig. S1), suggesting that the antibody itself had no adverse effect on cultured cells.

### PRL-3 chimeric antibody effectively inhibits the metastatic tumors formed by mouse cancer cells that express endogenous PRL-3

**Figure 2:**
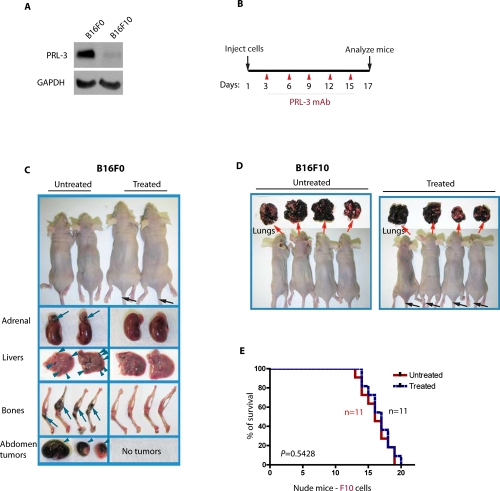
PRL-3 chimeric antibody effectively inhibits the formation of metastatic tumors formed by B16F0 cells that express endogenous PRL-3 **A.** Total cell lysates were prepared from B16F0 and B16F10 melanoma cells and analyzed by immunoblotting. Endogenous PRL-3 protein was readily detected in B16F0 but was almost undetectable in B16F10 cells. GAPDH was used as a loading control. **B.** A timeline of the therapeutic schedule of PRL-3 chimeric mAb administration. **C, D.** Mice were injected with 1 × 10^6^ B16F0 cells (C; n = 27) or B16F10 cells (D; n = 22), followed by the therapeutic schedule described in **B**. Organs were harvested, examined, and imaged on ~ day 17. **E.** Kaplan-Meier survival curves were used to compare treated and untreated B16F10 recipients. p < 0.05 was regarded statistically significant. n = numbers of mice per group.

To find a clinically relevant animal model to treat PRL-3-associated cancers, dozens of cancer cell lines for the expression of endogenous PRL-3 protein levels were screened by Western blot analysis. Ideal cell line pairs for our animal models should present contrasting levels of endogenous PRL-3 and should have the ability to induce metastatic tumors in mice within short timeframes. We found a pair of mouse melanoma cell lines B16F0 and B16F10 that fulfilled these criteria. Although B16F10 cells are naturally more metastatic than B16F0 cells, parental B16F0 cells express higher levels of endogenous PRL-3 protein than B16F10 cells (Fig. 2A), suggesting that the metastatic activity of B16F10 cells might be no longer PRL-3 dependent. When we employed an experimental metastatic assay [19] in which cultured cancer cells were introduced into the circulation of nude mice by lateral tail vein injection, both B16F0 and B16F10 cells rapidly formed multiple metastatic tumors in mice within 17 days. This aggressive *in vivo* metastasis model allowed testing of the differences in efficacy between ‘treated’ and ‘untreated’ groups shortly after antibody therapy. On day 3 post-injection of PRL-3 expressing B16F0 cancer cells, PRL-3 chimeric antibodies were administrated similarly via tail veins into the ‘treated’ mice, followed by two subsequent administrations of the antibody per week (Fig. 2B). ‘Untreated’ mice were administered with PBS (or control ascites). On day 17, the ‘treated’ mice appeared more active and healthier. The metastatic tumors in multiple tissues were dramatically reduced in ‘treated’ mice (Fig. 2C, right panel). The Kaplan-Meier survival analysis for these B16F0 recipients will be discussed in a later section. In a parallel experiment, the PRL-3 chimeric antibody had no effect in blocking metastatic tumors formed by B16F10 cancer cells that express low or undetectable level of PRL-3 protein. Hundreds of metastatic tumors were found in the lungs of all B16F10 recipients, and no obvious difference was seen between ‘untreated’ (Fig. 2D, left panel) and ‘treated’ mice (Fig. 2D, right panel). Furthermore, Kaplan-Meier survival analysis demonstrated that the PRL-3 chimeric antibody did not significantly extend the life-span for B16F10 recipients with a median survival of 16 days for ‘untreated’ and 17 days for ‘treated’ mice (Fig. 2E, *p* = 0.5428).

### PRL-3 chimeric antibodies effectively inhibit the metastatic tumors formed by human cancer cells that express endogenous PRL-3

**Figure 3:**
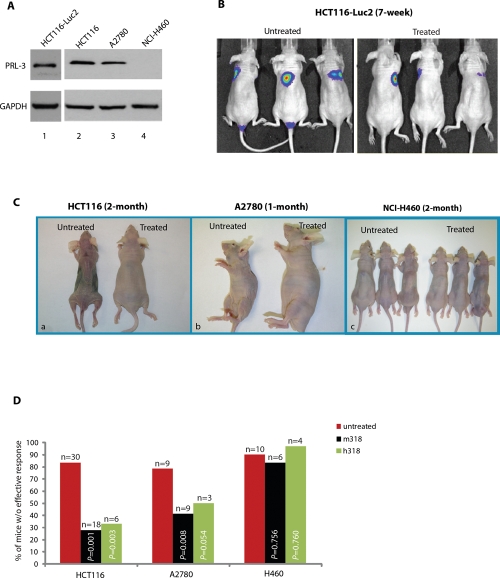
PRL-3 chimeric mAb inhibits the formation of metastatic tumors formed by A2780 cells and HCT-116 cells that express endogenous PRL-3 **A.** Total cell lysates were prepared from HCT116-*luc2*, HCT116, A2780, and NCI-H460 cancer cell lines. Endogenous PRL-3 protein was detected in HCT116-*luc2*, HCT116, and A2780 cells, but not in NCI-H460. **B.** On day 1, *nude* mice (n = 6) were injected with 1 × 10^6^ HCT116-*luc2* cancer cells and subsequently administered with PRL-3 chimeric mAb (treated, n = 3) or PBS (untreated, n = 3) on day 3, followed by biweekly intravenous administrations of the PRL-3 chimeric mAb or PBS, respectively, for 7-weeks. Both cancer cells and antibodies were injected via tail vein. IVIS Imaging System was used to track and monitor tumor development *in vivo*. **C.** On day 1, *nude* mice were injected with 1 × 10^6^ cancer cells and treated as described in **B**. Paired experiments (untreated/treated) were terminated when mice appeared moribund. Experiment durations are indicated on the top of each panel. **D.** Summary of results from the 101 mice used in this study, comparing the therapeutic efficacies of mouse PRL-3 (m318) or chimeric PRL-3 (h318) in *nude* mice injected with three different human cancer cell lines. Fisher's exact test was used to score the percentage of mice *without* effective treatment outcome. Red columns represent untreated mice, while black columns represent treated mice. *p* < 0.05 was regarded statistically significant. n = numbers of mice per group.

In addition to the B16F0 cell line, three additional high PRL-3-expressing cell lines were identified: HCT116, a human colorectal cancer cell line; HCT116-*luc2*, a HCT116 cell line that was established by transducing lentivirus containing the luciferase 2 gene (*luc2*) under the control of human ubiquitin C promoter; and A2780, a human ovarian cancer cell line (Fig. 3A lanes 1-3). A2780 has been reported as a PRL-3 positive cell line previously [20]. As a negative control, we identified a PRL-3 negative cancer cell line: NCI-H460, derived from human non-small lung cancer (Fig. 3A, lane 4). It is worth highlighting that regardless of PRL-3 expression, these four cancer cell lines could rapidly form metastatic tumors in *nude* mice within 1-2 months (Fig. 3, ‘untreated’ mice). The HCT116-*luc2* cell line together with Xenogen's *in-vivo* imaging system (IVIS) was used to monitor metastatic lung tumor formation in mice. Remarkably, the PRL-3 antibody could inhibit metastasis of HCT116-*luc2* cancer cells, with a clear reduction of metastatic lung tumors (in blue) in live imaging after 7 weeks of antibody therapy, compared to ‘untreated’ mice (Fig. 3B, right panel). Similarly, overt phenotypic differences were found between PRL-3 antibody ‘treated’ and ‘untreated’ mice at 2-month post-inoculation with HCT116 cells (Fig. 3C, a) and at 1-month post-inoculation with A2780 cells (Fig. 3C, b). PRL-3 chimeric antibody-treated animals appeared vibrant and healthy (up to 4-months), whereas all ‘untreated’ mice had lost weight and were moribund. In parallel, NCI-H460 (PRL-3 negative cells) recipients did not respond to PRL-3 antibody treatment (Figure 3C, c). To compare the effectiveness of PRL-3 antibody treatment on tumors formed by PRL-3 positive cell lines (HCT116, A2780) or PRL-3 negative cell line (NCI-H460) in *nude* mice, the occurrence of lung tumor metastases observed in ‘treated’ and ‘untreated’ groups were scored. Mice with less than 40% metastases reduction after treatment were regarded as lacking an effective outcome. Using this baseline, a clear beneficial effect was seen in mice inoculated with PRL-3 positive (but not negative) cells. The efficiency of both mouse- and chimeric-PRL-3 antibody therapy regimes were comparable (Figure 3D). Overall, these results further support the conclusion that the efficiency of the PRL-3 antibody treatment is tightly correlated with PRL-3 expression status of the cancer cells.

### NK cells are important in anticancer therapy

The efficacy of PRL-3 chimeric antibody in inhibiting PRL-3-tumor formation observed in *nude* mice suggest that T lymphocytes may not be essential in this antibody therapy since nude mice lack of mature T lymphocytes. We then investigated if Fc-binding, cytotoxic natural killer (NK) cells might play a role in the PRL-3 antibody therapy as NK cells are a subset of cytotoxic lymphocytes that constitute a major component of the innate immune system. To deplete the nude mice's NK cells, we pre-injected nud*e* mice with antibodies against asialo GM-1 NK cell-surface glycolipid, a procedure which has been shown to effectively eliminate NK cell activity [21, 22]. Thereafter, the above-mentioned procedures of the antibody therapy were repeated using B16F0 in these anti-asialo-GM1-injected *nude* mice. It was found that the therapeutic efficacy of our chimeric PRL-3 antibody was essentially lost in anti-asialo-GM-1-injected mice (Fig. S2). In support of the anti-tumor role of NK cells, anti-asialo-GM1-injected *nude* mice showed more severe tumors (in black)-bearing burden in lung, liver, adrenal, and testis than in mice without NK cell inhibition. Collectively, these results indicate the importance of the innate immune system and support a role for NK cells in mediating the intracellular antibody therapeutic response. Since NK cells have been demonstrated to have a role in human hematopoietic stem cell graft rejection [22], removal of NK cells may result in abolishment of graft rejection of NK cell activities, leading to tumor engraftment more successfully; indeed, we found ‘asialo-GM1- & PRL-3-treated’ mice were worse than ‘untreated’ mice in terms of tumor growth (Fig. S2).

### B-cells may play a role to mediate the therapeutic effects of PRL-3 antibodies

**Figure 4:**
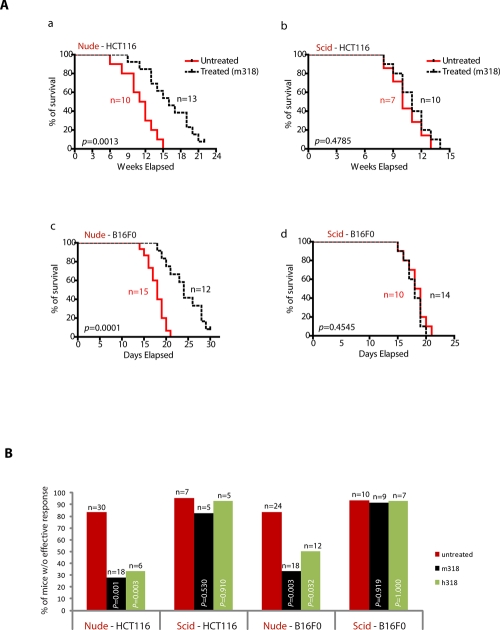
B-cells are important in mediating therapeutic efficacy of PRL-3 chimeric antibody **A.** Kaplan-Meier survival curves of treated and untreated HCT-116-injected *nude* and *scid* mice (a, b). Kaplan-Meier survival curves of treated and untreated B16F0-injected *nude* and *scid* mice (c, d). **B.** Summary of results from the 151 mice used in this study, comparing the results for therapeutic experiments in *nude* and *scid* mice (a-d). Fisher's exact test was used to score the percentage of mice without effective treatment indicated at Y-axis. Red columns represent untreated mice, while black or green columns represent mice treated with mouse mAb (m318) or chimeric mAb (h318), respectively. *p* < 0.05 was regarded statistically significant. n = numbers of mice per group.

To next address if B lymphocytes were also critical to our antibody therapy model, we compared the efficacy of antibody therapy between *nude* mice (lacking mature T-cells) and Severe Combined Immunodeficiency (*scid*) mice (lacking both mature T- and B-cells). These mice were i.v. injected with PRL-3 expressing cancer cells. In HCT116-injected *nude* mice (Fig. 4A, a: *Nude*-HCT116), Kaplan-Meier survival analysis revealed a statistically significant (*p* = 0.0013) in survival of ‘treated’ mice (16 weeks) compared to ‘untreated’ mice (11.5 weeks). In contrast, in HCT116-injected *scid* mice (Fig. 4A, b: *Scid*-HCT116), we observed that PRL-3 antibody therapy did not significantly (*p* = 0.4785) prolong the overall survival of *Scid*-HCT116 mice, with a median survival of 11-week for ‘treated’ and 10-week for ‘untreated’ mice. To further confirm the importance of B cells in responding to antibody therapies, another PRL-3-expressing cell line (B16F0) was used. In B16F0-injected *nude* mice (Fig. 4A, c: *Nude*-B16F0), Kaplan-Meier survival analysis showed that PRL-3 antibody treatment significantly increased median survival duration by 33% (24 days for ‘treated’, versus 18 days for ‘untreated’; *p* = 0.0001). Consistently, in B16F0-injected *scid* mice (Fig. 4A, d: *Scid*-B16F0), the antibodies had no effect in ‘treated’ mice, with both ‘untreated’ and ‘treated’ groups showing similar median life-span (18.5 and 18 days respectively; *p* = 0.4545). As before, the therapeutic efficacy was independent of antibody species, as similar results were obtained using either mouse or chimeric PRL-3 antibodies (Fig. 4B, black or green column). Collectively, the results from these genetic mouse models suggest that PRL-3 antibody therapeutic efficacy against PRL-3 metastatic tumor formation is dependent on host B-cell but not T cells. Other than antibody production, we hypothesized that B-cell may have additional functions; such as the secretion of unknown factor(s) that could facilitate the action of antibodies.

### PRL-3 antigen is insignificantly present at the cell surface

**Figure 5:**
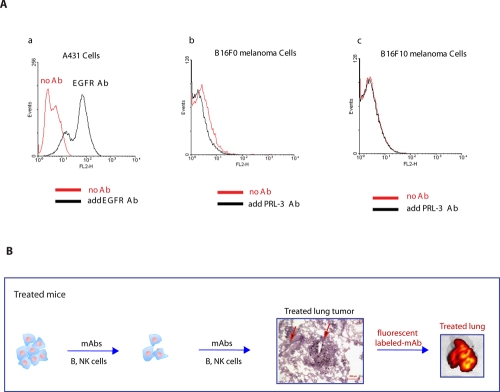
PRL-3 is not detectable at the cell surface, but accumulates promptly at sites of cancer metastasis in vivo **A.** a. A peak-shift observed in A431 cells incubated with EGF-receptor antibody (black line) compared to A431 cells without incubation of EGF-receptor antibody (red line). b. No peak-shift was observed in B16F0 cells incubated with PRL-3 mAb (black line) and without Ab (red line). c. No peak-shift was observed in B16F10 cells incubated with PRL-3 mAb (black line) and without Ab (red line). **B**. *Nude* mice were injected with B16F0 cancer cells, ‘treated’ mice were followed by the therapeutic schedule in Fig. 2B. Labeled PRL-3 antibodies were intravenously injected 1 hr before IVIS live imaging at the end of experiment. A strongly fluorescent labeled lung was evident in ‘treated’ mice. We hypothesize that early delivery of the antibody will allow persistent attack of cancer cells to prevent them from further progression, resulting in micro-metastases in ‘open’ stages. As such, fluorescent labeled PRL-3 antibody can access and bind to metastatic lung tumors in such ‘treated’ mice. Scale bar: 200 μm. Red arrows indicate blood vessels.

A possible mechanism of PRL-3 antibody action could be its binding of PRL-3 encoded cell surface antigen(s), thereby triggering a B-cell and/or NK-cell dependent elimination of the PRL-3 expressing cell. However, to date, no reports describe a cell surface localization of PRL-3. To address the possibility of PRL-3 antibody binding its antigen on the cell surface, we used a FACS assay routinely used in cell surface labeling. As a positive control, anti-EGFR antibody binding of the EGFR-overexpressing human epidermoid carcinoma A431 cell line was used for this assay. Incubation of A431 cells with anti-EGFR antibodies caused a distinct peak-shift in the FACS assay (Fig. 5A, a). However, no peak shift was observed in either B16F0 (PRL-3 positive) or B16F10 (PRL-3 negative) cells incubated with or without anti-PRL-3 antibody (Fig. 5A, b-c). These results implied that extracellular PRL-3 protein, if any, was unlikely to be the major cause of antibody binding. However, since immune system constantly battles invaders, such as bacteria and viruses, and cancer cells *in vivo*, the cancer cells could be destroyed and lysed to expose their intracellular proteins to the immune system and produce a localised inflammatory reaction to trigger cell death. If so, the same explanation may apply to PRL-3 and other intracellular oncoproteins as well.

### In-Vivo Imaging System (IVIS) live imaging-based working models

To investigate if PRL-3 mAb can bind and reach to PRL-3 metastatic tumors, we used fluorescent labeled PRL-3 mAb to track sites of metastatic tumors. It was observed that the strong fluorescent labeled PRL-3 antibodies were concentrated at the lung filled with PRL-3 metastatic tumors (Figure 5B), suggesting that the fluorescent labeled antibodies can arrive at the metastatic lung tumors *in vivo*. With the current clinical practice of assessing whether the cancer is Her2-positive before considering the Herceptin antibody therapy [23], a similar approach can be used for antibodies which target the PRL-3 intracellular oncoprotein to prevent further spreading or relapse in PRL-3 positive cancer patients. Other than using PRL-3 mouse antibody to perform IHC test on cancer biopsy in order to identify PRL-3 positive patients, the IVIS live imaging system may serve as another method to screen PRL-3 positive cancer patients for the treatment using the PRL-3 chimeric antibody.

### PRL-3 chimeric antibody is likely to have broad applications in inhibiting PRL-3 positive cancers

**Figure 6:**
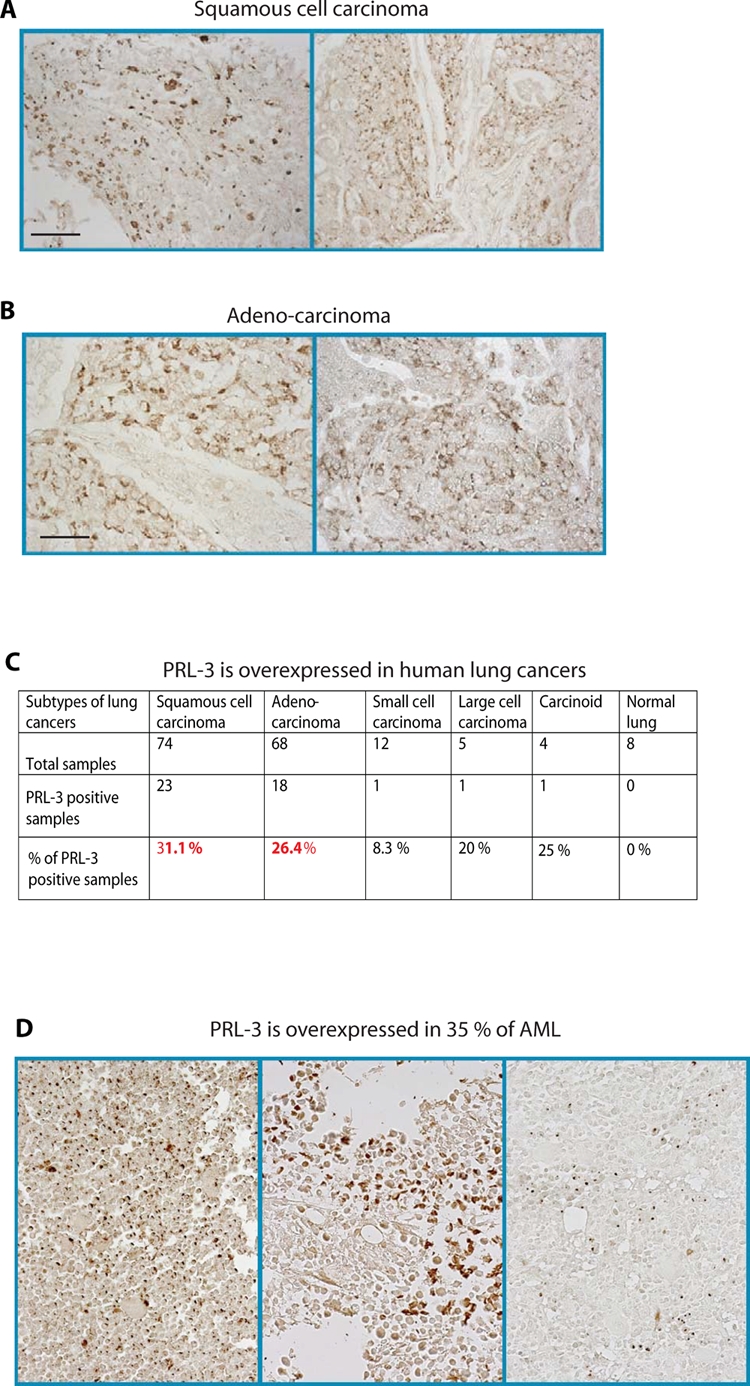
PRL-3 protein is upregulated in lung cancers and AML **A, B.** Representative immunohistochemistry (IHC) staining of PRL-3 expression in lung squamous cell carcinoma (A) and lung adenocarcinoma (B). Scale bar: 100 μm. **C.** Summary of the percentages of PRL-3-positive lung cancers as detected with IHC, grouped according to cancer subtypes. **D**. In AML bone marrow samples were examined by IHC, 24 out of 69 (35%) showed PRL-3 expression. Three representative images are shown.

Genes specifically upregulated during tumor formation but poorly or not expressed in host tissues are particularly promising as tumor-specific targets. PRL-3 was reported to be upregulated in multiple human cancers [24, 25] and was not detected in most of the mouse tissues (Fig. S3A). Therefore, PRL-3 fulfills the criteria to be validated as a biomarker and a target for anticancer therapy. We anticipate that the PRL-3 antibodies are likely to have broad applications to block multiple types of PRL-3 positive cancer spreading, especially in some lethal malignancies such as lung cancers and *acute myeloid leukemia* (AML) that often relapse within short timeframes. Amongst lung cancers, we found PRL-3 is overexpressed in 31% of squamous cell carcinoma and 26% of adenocarcinoma (Fig. 6A-C), two main subtypes of highly recurrent non-small cell lung carcinoma comprising 80% of human lung cancer. We also found that PRL-3 is overexpressed in 35% (24 out of 69 cases) of AML bone marrow samples (Fig. 6D).

## DISCUSSION

PRL-3 is upregulated in numerous types of human cancers and is involved in their metastasis [10, 24, 25]. An evidence-based concept is hereby proposed for a possible approach in targeting intracellular oncoproteins with antibody therapy. Using different mouse models and cancer cell lines, we consistently demonstrated herein that antibodies to intracellular PRL-3 protein could specifically exert therapeutic effects. It is anticipated that the PRL-3 chimeric antibody is likely to have broad applications in blocking the progression of different types of PRL-3 positive cancers, particularly in malignancies such as lung cancers and *acute myeloid leukemia* (AML) that often relapse within short time frames. Therefore, therapeutic effects will be more apparent after treatment in acute cancers, when compared with ‘untreated’ PRL-3 positive patients.

Currently, our understanding of how PRL-3 antibodies inhibit PRL-3-positive tumors *in vivo* is limited. Despite previously proposing several possible mechanisms of action based on *in vitro* data on how antibodies may access to cancer cells [16], the precise mechanism(s) behind these findings is still an active area of research. However, it is of important to note that the precise mechanism(s) of anti-HER2/neu antibody therapy is not yet understood even though the anti-HER2/neu antibody has been used clinically for more than a decade for treating breast cancers [23]. We demonstrate a lack of PRL-3 antibody cytotoxic effect *in vitro*, even at high antibody concentrations (Fig. S1), suggesting that using *in vitro* cell culture systems to elucidate the mechanism of intracellular antibody therapy is not representative because the cell culture system is too simplified due to the limitation of a single cell type grown *in vitro*, lacking the interplay of complex *in-vivo* systems – omission of critical interactions with the tumor microenvironment. Nevertheless, from the results in this study, our reproducible observations here and elsewhere indicate that intracellular oncoproteins are viable targets for anticancer therapy. We highlight the following points to be considered before translating these results into clinical applications: *1) Antibody treatment efficacy is tightly correlated with the expression status of its antigen in cancer cells*. We demonstrated here that the PRL-3 chimeric antibody could successfully block the formation of metastatic tumors derived from several cancer cell lines (B16F0, HCT-116, and A2780) that express intracellular PRL-3 phosphatase. The inhibition was specific as the antibody had no effect in blocking the formation of metastatic tumors derived from other cancer cell lines (B16F10, H460) that did not express PRL-3. Our previous results had demonstrated that mouse PRL-3 antibody had no effect in inhibiting metastatic lung tumors formed by CT26 mouse colon cells, another PRL-3 negative cancer cell line [16]. Together, the data support the notion that the efficiency of both mouse and chimeric PRL-3 antibody treatment is tightly correlated with PRL-3 expression status of the cancer cells. If the metastatic property of cancer cells was not associated with PRL-3 expression (such as B16F10, H460, CT26 cell lines), the administration of PRL-3 mAb would have no effect in blocking tumors formed by these PRL-3 negative cells. Furthermore, we previously [16] had shown that PRL-1 mAb specifically blocks PRL-1 (but not PRL-3) metastatic tumors; while PRL-3 mAb specifically blocks PRL-3 (but not PRL-1) metastatic tumors. This is especially remarkable because PRL-1 and PRL-3 share high homology (76%) in protein sequence. Collectively, these results imply that PRL antibody therapy is highly specific to its antigen and does not involve cross-reactivity with other non-specific cell surface proteins. *2) Not all intracellular oncoproteins are viable targets for antibody therapy*. Desirable anti-cancer therapeutic agents should specifically target cancer cells while leaving normal tissues unharmed. It should be emphasized that the PRL-3 chimeric antibody therapy has little detectable side effect in *nude* mice as PRL-3 expression in normal tissues is not ubiquitous. We showed that PRL-3 protein was detected in only a few organs such as the spleen, brain, and pancreas (Fig. S3A). In contrast, PRL-2 is ubiquitously expressed in most of the mouse tissues (Fig. S3B). As expected, PRL-2 antibody therapy to PRL-2 expressing cancers was unsuccessful (Fig. S3C), and PRL-2 antibody-treated mice died 1-week earlier or showed worse outcome than ‘untreated’ mice, likely due to the side effect of anti-PRL-2 antibody causing normal PRL-2-expressing tissues to be targeted by the PRL-2 antibodies. Thus, the choice of a good therapeutic target should be tumor-specific to avoid harming host normal tissues. *3) NK cells of the innate immune system are involved*. To understand if innate immune system was involved in antibody therapy, we intravenously injected anti-asialo-GM1 antibody via tail vein into *nude* mice to deplete NK cells, which are a type of cytotoxic lymphocytes that constitute a major component of the innate immune system. In the absence of NK cell activity, we found that anti-PRL-3 antibody lost therapeutic efficacy. Furthermore, tumor-engraftment was dramatically enhanced (Fig. S2), indicating that NK cells normally play a critical role in graft-rejection for implantation of foreign cells. Evidence also suggests that NK cells play an important part in the destruction of incipient tumors [21]. *4) Antibody-dependent cellular cytotoxicity (ADCC) may also be involved*. The best characterized mechanism of antibody therapy is antibody-dependent cellular cytotoxicity (ADCC). In ADCC, antibodies bind to specific cell surface antigens and trigger an Fc-mediated immune response involving cytotoxic CD8 T-cells, complement activation, and/or NK cell activity. Although we did not observe any peak-shifts in our FACS analysis of PRL-3 cell surface antigen in neither PRL-3-expressing B16F0 cells nor PRL-3 low-expressing B16F10 cells, we could not rule out a possibility that these cancer cells were under abnormal inflammatory pressure, which may cause the destruction of cancer cells releasing their intracellular protein. This enables the antigen-antibody reaction to trigger specific immune response with lymphocytes removing these cancer cells. Alternatively, *in vivo*, cancer cells are under hypoxic stress steps and serum deprivation, conditions that arrest cells at G and G phases [26]. It is possible that these conditions may cause release of intracellular antigens for antibody recognition. If so, other intracellular oncoproteins may also encounter the similar situations, and could therefore be similarly targeted with antibody therapy. *5) The adaptive immune system is important in eliciting mAb therapeutic effect*. We observed a therapeutic effect only in *nude* mice but not in *scid* mice in our antibody therapy experiments. The major differences between *nude* and *scid* mice are that *nude* mice are T-cell deficient, but have functional IgM antibodies and B cells, whereas *scid* mice have no functional adaptive immune system including B-cells, T-cells, IgM and other antibodies. Both *nude* and *scid* mice, however, have intact innate immune systems including normal NK cell and complement activity. The positive response seen only in *nude* (but not in *scid*) mice, indicates that mature B cells (but not T cell), and possibly IgM/serum antibodies, are more important and might co-ordinate with the innate immune system in generating the anticancer response observed here. Since the PRL-3 antibodies here were exogenously introduced into mice, we hypothesize that the requirement of B-cells for antibody activity might be due to an alternative role for B-cell in the antibody response, possibly via secretion of unidentified factor(s) that modulates the host response. Herein, we emphasize that the intricate interplay of innate and acquired immune system is crucial for the anticancer efficacy of a chimeric antibody targeting intracellular PRL-3 oncoprotein*. 6) Antibody therapy against intracellular oncoprotein is clinically relevant*. To generate an aggressive cancer model for antibody treatment, we directly injected 1 million cancer cells into mouse blood circulation. Given that the total blood volume in *nude* mouse is 8% of its body weight (8% of ~19g = 1.5ml), this translates to the introduction of approximately 6.7 × 10^5^ cancer cells/mL of blood. Remarkably, despite such a high cancer cell concentration in blood, we observed overt therapeutic benefit, despite delaying antibody treatment until 3-day post-cancer cell injection. In light of this, we hypothesize that a few injections of chimeric antibody could significantly reduce the recurrence rate of operable tumors, as the PRL-3 antibodies continue to act on any remaining circulating cancer cells [27] by removing non-visible incipient tumors after surgical resection.

Herein, we have conducted the first chimeric antibody study in targeting an intracellular oncoprotein (PRL-3, in this case) for cancer therapy in mice. We also recently proposed that other intracellular oncoproteins could also be targeted with antibody therapy and/or vaccination [28]. The lack of observable side effects in *nude* mice upon PRL-3 antibody therapy further alludes to its potential clinical benefits. As with the current clinical practice of assessing the Her2-status of cancers before considering Herceptin antibody therapy [29], a similar approach could be used for antibody targeting PRL-3 intracellular oncoprotein to circumvent further spreading or relapse in PRL-3 positive cancer patients. Importantly, our data prompts an evaluation of a wide spectrum of tumor-specific intracellular oncoproteins as possible targets for anti-cancer mAb therapy, thus realizing the full potential of antibodies as ‘magic bullets’.

## MATERIALS AND METHODS

### Generation of specific PRL-3 human/mouse chimeric mAb (clone #318)

To generate PRL-3 chimeric mAb, total RNA was extracted from 6 × 10^6^ hybridoma cells (clone #318) [17] using the RNeasy Mini Kit (QIAGEN, Cat #74104). The RNAs were then reverse-transcribed into cDNA using SuperScript II RNase H (Invitrogen, Cat 18064-014). The resulting total cDNAs were used as templates to generate the ‘universal variable region’. The Ig-Primer Sets (Novagen, Cat #69831-3) were designed for amplification by PCR (95°C-4°C-72°C, 30 cycles) to specifically amplify of immunoglobulin (Ig) variable region of light- and heavy-chain cDNAs from mouse sources. The PCR fragments were cloned into the PCRII-TOPO-Vector with a TA cloning kit (Invitrogen, Cat #45-0640). Appropriately designed oligonucleotide primer sets enable these variable region of light- and heavy-chain cDNAs to be cloned into the respective sites of a human IgG1 constant region expression vector-pCMV-human IgG1 [18] to join the mouse variable region with the human IgG1 constant region. The complete construct was transiently transfected into 293T cells cultured in media supplemented with ultra-low IgG FBS (Gibco, 16250-078). The chimeric mAb was subsequently harvested from the culture supernatant and concentrated up to 40 folds with centrifugal filter devices (Millipore, Cat #UFC900596). The chimeric mAb was then tested for its specificity by indirect immunofluorescence (IF) and Western blot analysis.

### Cell lines and cell culture

HCT116 (CCL-247) human colorectal carcinoma cell line, NCI-H460 (HTB-177) human non-small lung cancer cell line, A431 (CRL-1555) human epidermoid carcinoma cell line, B16F0 (CRL-6322), and B16F10 (CRL-6475) mouse melanoma cell lines were purchased from the American Type Culture Collection (ATCC, Manassas, VA). A2780 (Cat #93112519) human ovarian cancer cell line was purchased from ECACC, UK. Cells were grown in appropriate media recommended by the suppliers.

### Western blot analysis

Generation of mouse PRL-3 monoclonal antibody and Western blot procedures have been described previously [17]. GAPDH antibody was from Millipore (Bedford, MA). Donkey anti-human HRP-conjugated secondary antibody (Cat #709035149) was obtained from Jackson ImmunoResearch Laboratories (West Baltimore, PA).

### Experimental Metastatic Assay in mice [20]

1 × 10^6^ cancer cells were injected into the circulation of eight-week old *nude* mice (Jackson Labs, USA) via the tail vein on day 1. Chimeric PRL-3 mAbs (h318) or mouse PRL-3 mAbs (m318) were subsequently injected into the tail vein, with the first antibody administration was carried on day 3 post-cancer cell injection (the latest time we can delay for treatment), followed by two administrations per week. For control untreated group, PBS was administrated via tail vein. All animal studies were approved by the Institutional Review Board (IRB) of the Institute of Molecular and Cell Biology (IMCB), in strict compliance with rules and policies of the Animal Facility Center of The Agency for Science, Technology and Research (A* STAR), Singapore.

### Depletion of NK cells

Anti-asialo GM1 anti-serum (raised in rabbit) was purchased from Wako Pure Chemical Industries, Ltd (Osaka 540-8605, Japan). The anti-asialo GM1 anti-serum (50 μl) was injected into the circulation of eight-week old *nude* mice (Jackson Labs, USA) via the tail vein 24 h before 3 × 10^5^ cancer cells were injected into the circulation of the *nude* mice. Subsequent steps of antibody therapy were carried out as described earlier.

### Antibody labeling and IVIS live imaging

HCT116-*luc2* Bioware Ultra cell line, obtained from Caliper Life Sciences, Inc. (Hopkinton, MA), was established by using HCT116 human adenocarcinoma cells (ATCC, CCL-247^T^) stably transduced with lentivirus containing luciferase 2 gene under the control of human ubiquitin C promoter (pGL4 *luc2*). 1 × 10^6^ HCT116-*luc2* cancer cells were injected into tail veins of 8-week old nude mice. Antibody was then injected into ‘treated’ mice via tail veins on day 3, follow by two antibody injections per week. After 7-week treatment, purified PRL-3 antibody (by protein G/A bead) was labeled using a CF750 Dye Antibody Labeling Kit from Caliper Life Sciences, Inc. (Hopkinton, MA). Labeled antibodies were injected via tail vein 1 hr before live imaging. Mice are injected by an intraperitoneal route with a luciferin solution (200 × stock solution 30 mg/ml in PBS, dose of 150 mg/kg) that is allowed to distribute in conscious animals for about 5-15 minutes. The mice were then placed into a clear plexiglass anesthesia box (supplied with 2.5-3.5% isofluorane) that allows unimpeded visual monitoring of the animals using IVIS® Spectrum Imaging System 3D Series to track and monitor tumor development *in vivo*. The results between ‘treated’ verse ‘untreated’ mice were determined.

### FACS analysis

The human epidermoid carcinoma cell line A431 was grown in DMEM with high glucose (4.5g/L), supplemented with 10% FBS and 5% antibiotic. B16F0 and B16F10 cells were grown in RPMI, supplemented with 10% FBS and 5% antibiotics. Cells (5 × 10^6^) were dislodged from the dishes with non-enzymatic pre-warmed cell dissociation solution (Sigma, Cat #c-5914) and transferred to 5 ml polystyrene tubes and washed once with complete medium. The cells were then incubated with 1 μl EGFR (Genetech, USA) or 5 μl PRL-3 mouse primary antibodies in 100 μl of complete medium for 1 h at room temperature (RT). Cells were agitated every 15 minutes to prevent clumping. Cells were then washed twice with complete medium and incubated for 1 h at RT with goat anti-mouse AlexaFluor 546 antibody (Invitrogen, USA), washed, and re-suspended in 1 ml complete medium prior to analysis using BD FACS Caliber. Raw data was processed using WinMDI ver2.8 software.

### Histopathologic Analyses Using Immunohistochemistry (IHC)

Human lung cancer tissue arrays 009-01-004, CC00-01-006 and CC04-01-CC04 were purchased from Cybrdi, Inc. (Rockville, MA). Human AML bone marrow samples were obtained from the National University Hospital-National University of Singapore (NUH-NUS) Tissue Repository with approval of the Institutional Review Board (IRB) of NUH-NUS for research use. The use of all human tissue samples including commercial samples were approved by IRB of IMCB. Dako EnVisionTM Systems K 1395 (Carpinteria, CA) was used to perform IHC analysis [16,17].

### Statistical analysis

The Kaplan-Meier method was used to compare survival time between ‘treated’ and ‘untreated’ mice groups. Fisher's exact test was used to analyze the association of treatment response to treatment groups. A *p* value < 0.05 was considered statistically significant. Graphpad Prism4 software package (La Jolla, USA) was used for all statistical calculations.

## Supplementary Figures


