# Supplementary material for: Engineering the First Chimeric Antibody in Targeting Intracellular PRL-3 Oncoprotein for Cancer Therapy in Mice
Source: Oncotarget. 2012 Feb 27;3(2):158–71. doi: 10.18632/oncotarget.442 (PMC3326646; doi:10.18632/oncotarget.442)
Supplement: Supplementary file 1 [file oncotarget-03-158-s001.pdf]

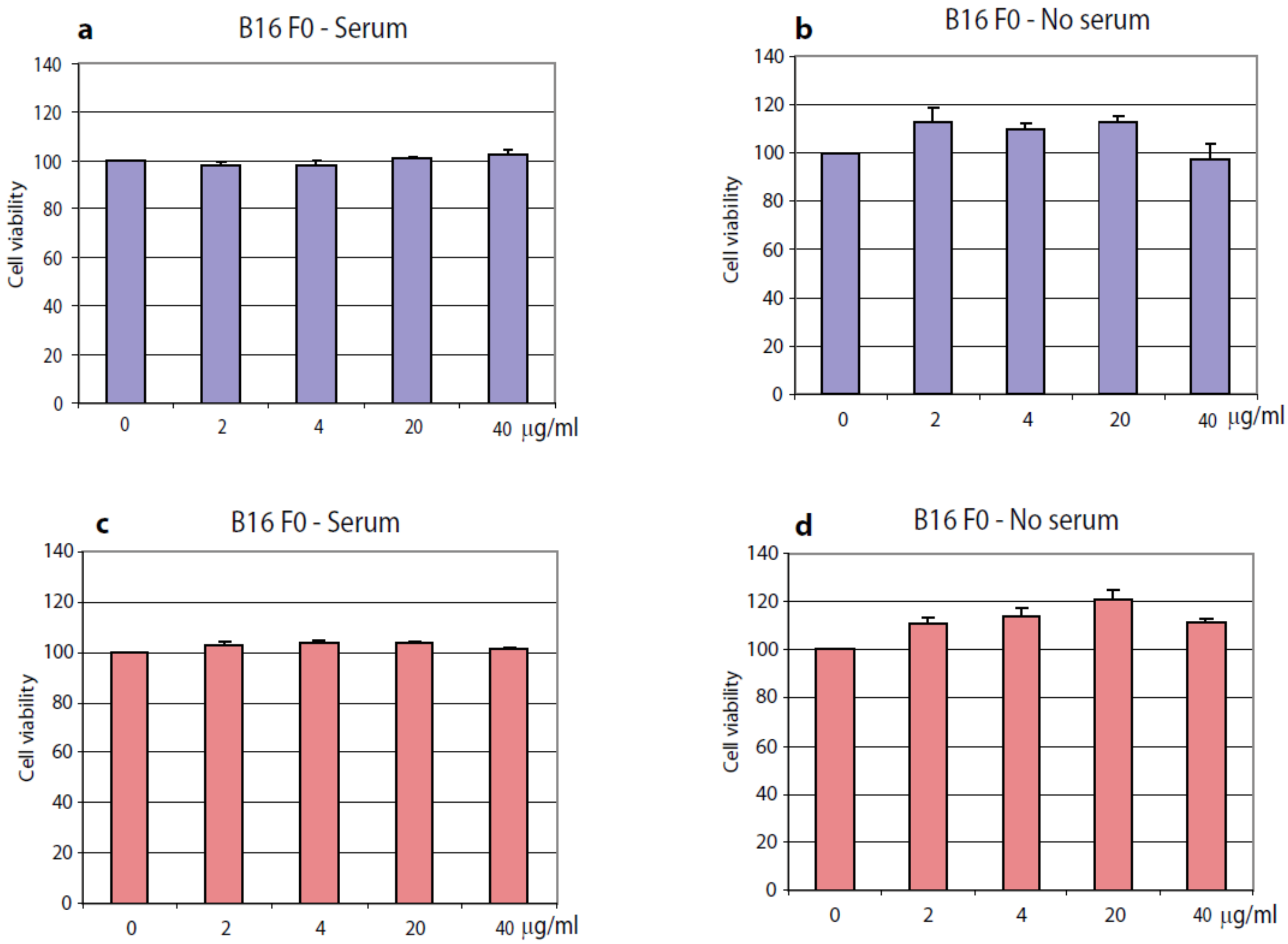

Figure S1. **PRL-3 chimeric antibody was evaluated for the 50% cell inhibitory (cytotoxic) concentration (IC50).** In this test, 96-well tissue culture plates are seeded with B16 mouse melanoma cells and exposed to varying concentrations of the antibodies [amount of chimeric antibody (h318) was indicated at X-axis)] in a CO2 incubator at 37°C with (a, 24hr, c, 48hr) or without serum (b, 24hr, d, 48hr), at which time 20% crystal violet is added and the degree of colour intensity indicating viable cell number is determined spectrophotometrically; an IC50 is determined by regression analysis. The cell viability was not affected up to (40 µg/ml) for 48 hrs. The cell numbers (x10^6) were indicated at Y-axis. The experiments were performed three times to confirm the results (mean ± SD).

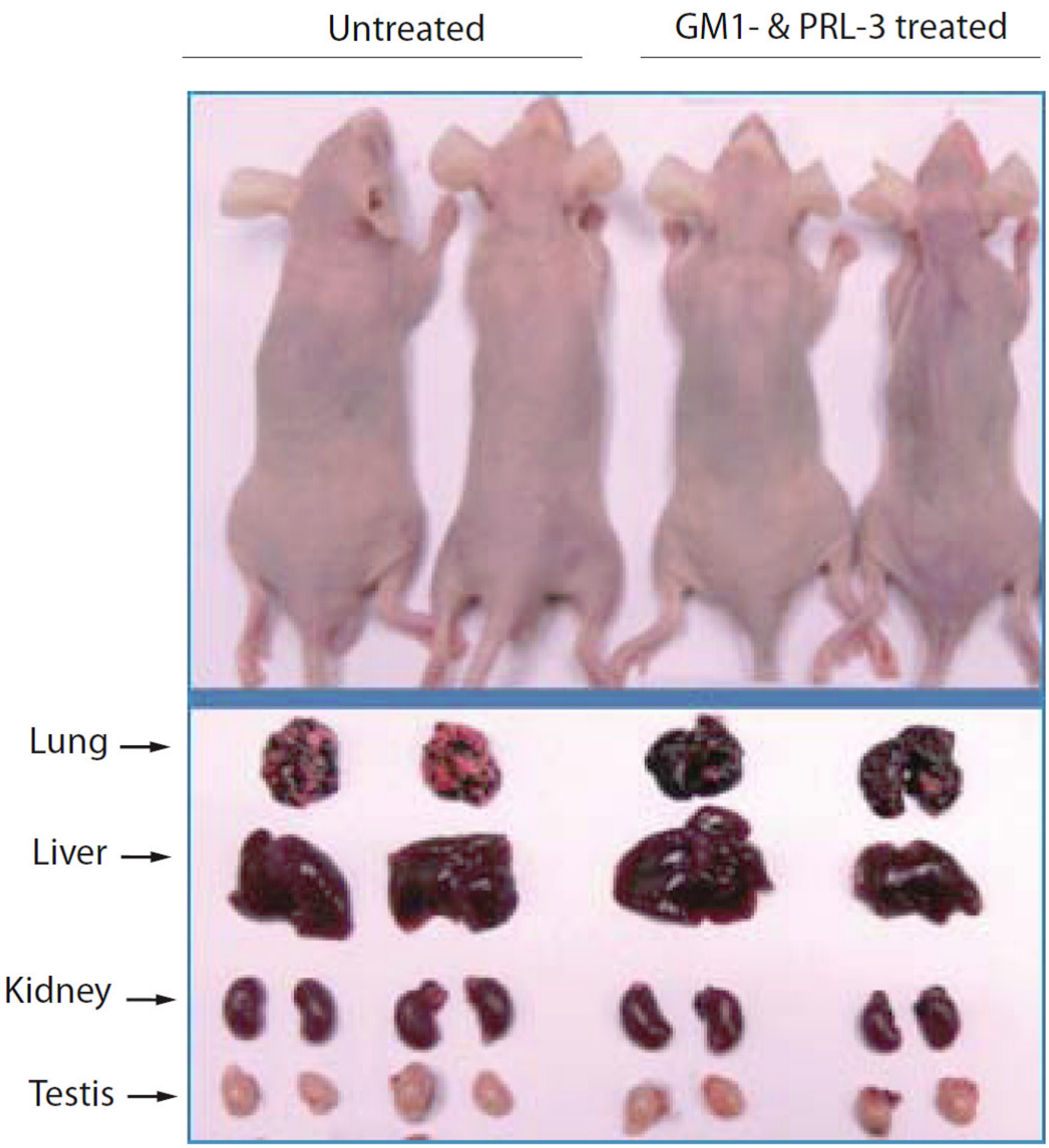

Figure S2: NK cells in Innate Immune system are involved in the therapy. *Nude* mice were injected (n=2) and un-injected (n=2) with Anti-asialo GM1 anti-serum 24 hrs before the experiment. On day 1, all mice were injected with $1x10^6$ F0 cells via tail vein, followed by two intravenous administrations of the PRL-3 chimeric mAb per week (day 3, 6, 9, 12, 15) in GM1 injected mice. On day 18, the therapeutic efficacy was examined. GM1 injected *nude* mice (regardless PRL-3 antibody treatment or not) showed more severe tumors (in black)-bearing burden in the lung, liver, adrenal, testis, and bone than GM1 untreated mice, regardless PRL-3 mAb treated or untreated.

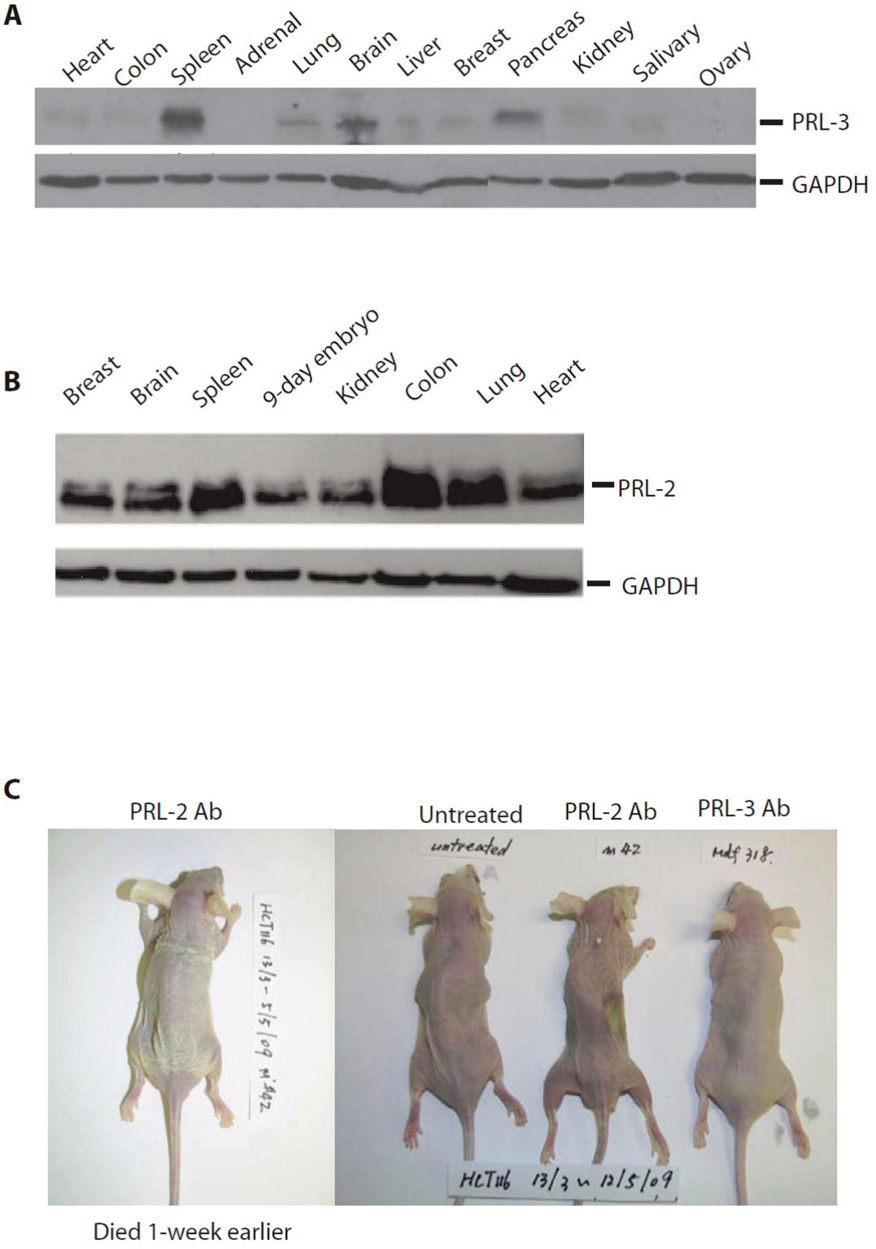

Figure S3: The outcome of the antibody treatment is highly associated with tissue expression patterns of the targets. **A, B.** The protein expression patterns of PRL-3 and PRL-2 in normal mouse tissues examined by western blot, GAPDH was used as a loading control. **C.** HCT-116 (PRL-2 positive cell line) recipients failed to respond to PRL-2 antibody therapy, most likely due to the fact that PRL-2 is ubiquitously expressed in most of mouse tissues.
